# A neural flexible PID controller for task-space control of robotic manipulators

**DOI:** 10.3389/frobt.2022.975850

**Published:** 2023-01-04

**Authors:** Nguyen Tran Minh Nguyet, Dang Xuan Ba

**Affiliations:** ^1^ Faculty of Electrical and Electronics Engineering, HCMC University of Technology and Education (HCMUTE), Ho Chi Minh City, Vietnam; ^2^ Department of Automatic Control and Smart Robotic Center, HCMC University of Technology and Education (HCMUTE), Ho Chi Minh City, Vietnam

**Keywords:** intelligent controller, robotic, manipulators, PID controller, neural network

## Abstract

This paper proposes an adaptive robust Jacobian-based controller for task-space position-tracking control of robotic manipulators. Structure of the controller is built up on a traditional Proportional-Integral-Derivative (PID) framework. An additional neural control signal is next synthesized under a non-linear learning law to compensate for internal and external disturbances in the robot dynamics. To provide the strong robustness of such the controller, a new gain learning feature is then integrated to automatically adjust the PID gains for various working conditions. Stability of the closed-loop system is guaranteed by Lyapunov constraints. Effectiveness of the proposed controller is carefully verified by intensive simulation results.

## 1 Introduction

Today, the great development of science and technology has created a premise for scientific research to develop to a new level in which the field of robotics has being chosen to be the leading industry by many countries. To promote science and technology backgrounds, intelligent robots in the industrial application are starting to prosper strongly, attracting many research experts. To control robot moving safely to desired positions with obstacles, collision avoidance and path planning were matters of concern. In recent years, various strategies have been studied for collision avoidance control purpose. The basic idea behind the collision avoidance algorithms is to design a proper controller which can result in a conflict-free trajectory. Path selection methods are the one of several techniques to avoid obstacles. It uses off-line/on-line algorithms to produce a curve that connects the starting and target points with a predefined initial position, velocity and acceleration. For example, an online trajectory generation algorithm called Ruckig considered third-order constraints (for velocity, acceleration, and jerk), so the complete kinematic state could be specified for waypoint-based trajectories (Berscheid and Kroeger, 2021). The smooth trajectory based on method combining of fourth and fifth order polynomial functions was presented in (Boscario et al., 2012) in which, the outcome of the method was the optimal time distribution of the *via* points, with respect to predefined objective function. After that, the joint based controller might use the inverse kinematic to solve the desired joint angular. Early collision avoidance approaches concentrated on the static obstacles handling by the sensor-based motion planning methods (Borenstein and Koren, 1991), using nearness diagram navigation to successfully navigate in troublesome scenarios (Minguez and Montano, 2004) and using trajectory planning algorithms to avoid obstacles (Shiller, 2015). In reality, many techniques have been proposed to cope with moving obstacles. For instance, a reactive avoidance method incorporating with a non-linear differential geometric guidance was presented in (Mujumdar and Padhi, 2011) and a collision avoidance algorithm based on the potential fields was proposed in (Huang et al., 2019). It can be seen that in normal applications of robotic manipulators, the controllers were designed in the joint space in which it requires exact inverse kinematic computation as well. Non-etheless, complex internal dynamics and external disturbances coming from divergent working conditions are main obstacles hindering development of excellent controllers.

To realize control objectives of the robots in real-life missions, simple proportional-integral-derivative (PID) controllers are priority options (Bledt et al., 2018), (Wensing et al., 2017) due to simple design. If the proper control gains were found, the high control outcomes could be obtained (Park et al., 2015), (Ba and Bae, 2020). A lot of research have been then studied to improve the performance of the PID controllers using intelligent approaches such as evolutionary optimization and fuzzy logic (Astrom and Hagglund, 1995). The methods exhibited promising control results thanks to using both online and offline sections (Tan et al., 2004). The off-line control one could flexibly select the proper PID parameters based on the system overshoot, settling time and steady-state error, while the on-line one would adopt the operating control errors to adjust fuzzy logic parameters to re-optimize the system, improving the system quality significantly. However, the tuning methodology of fuzzy logic controllers is mostly based on experiences of operators (Juang and Chang, 2011). Another series of the intelligent control category was based on the biological properties of animals in which a genetic algorithm was combined with a bacterial foraging method to simulate natural optimization processes such as hybridization, reproduction, mutation, natural selection, etc., (Cucientes et al., 2007). This evolution could deliver the most optimal solution. That the solving process requires a large number of samples and takes a long-running time limits its application. Recently, tuning PID control parameters using neural networks has become an effective approach with many contributions (Kim and Cho, 2006), (Neath et al., 2014). The conventional PID one itself is a robust controller (Thanh and Ahn, 2006). The learning ability integrated to the controllers makes it flexible to the working environment (Ye, 2008). Lack of an intensive consideration of learning rules in steady-state time could make the system unstable in a long time used (Ba et al., 2019), (Ye, 2008), (Rocco, 1996).

To further improve the control performance, internal and external dynamics of robots need to be compensated during working processes. To this end, classical methods could be employed based on accurate mathematical models of the robots (Craig, 2018), (Zhu, 2010). Good control results were exhibited using such the conventional approaches, but it is not easy to extend the control outcome to complicated robot structures. Intelligent modeling methods could be adopted to increase applicability of the controllers to various robots in different working environments (Karayiannidis et al., 2016), (Gao et al., 2022). Excellent control performances were accomplished with the intelligent control approaches. However, convergence of the learning process is still not explicitly proven (He et al., 2020), (Wang et al., 2020). To support this kind of theoretical drawback, linear leakage functions were integrated the estimation phases of the network operation. However, this term could be slowdown the overall learning performance. Hence, advanced learning behaviors for the network need to be extensionally studied.

In this paper, an intelligent direct PID controller is proposed for position-tracking control in task space of robotic manipulators. Without using inverse kinematics, the operator just needs to input the desired position value, the controller will calculate and give the desired control position to the robot by itself (Craig, 2005; Ba and Bae, 2021; Ba et al., 2021). This process will be of great help since, in practice, there are quite few robots with quite complex hardware structures that make the inverse kinematics calculation difficult. The more degrees of freedom a robot has, the more difficult the calculation process, requiring more time and effort. The proposed controller is built based on a conventional PID framework. A non-linear neural network is then employed to eliminate internal/external disturbances during the working process. To increase the adaptive robustness of the controller, a new gain learning rule is integrated to flexible tune the PID gain for different working conditions.

Outline of the paper is structured as follows. Section 2 discusses system modeling and problem statements. Section 3 presents design of the proposed controller. Section 4 analyzes verification results. The paper is then concluded in Section 5.

## 2 System modelling and problem statements

Behaviors of a general robotic manipulator can be presented in the following form (Craig, 2018), (He et al., 2020):

$$M\left(q\right)\ddot{q}+C\left(q,\dot{q}\right)+G\left(q\right)+\tau_{f}+\tau_{d}=\tau ,$$ (1)

where $q,\;\dot{q},\;\ddot{q}$ are respectively vectors of joint position, velocity, and acceleration, $M\left(q\right)$ is the mass matrix, $C\left(q,\dot{q}\right)$ is the centrifugal-Coriolis moment, $G\left(q\right)$ is the gravitational moment, $\tau_{f}$ is the frictional moment, $\tau_{d}$ stands for external disturbances, and τ is the actuator moment or control signals.

Remark 1: the control objective of this paper is to find out a proper control signal ( τ ) to control position of the end-effector of the robot following a desired profile. To accomplish this task, we can use inverse kinematics (IK) to compute desired joint positions from the end-effector reference signals. However, it is not trivial to find solutions of complicated robots. To avoid this shortcoming, we can apply direct control algorithms without caring of the IK problem. Hence, one needs consider dynamic model (1) in the task space as follows (Craig, 2018):

$$\ddot{x}=J\left(q\right){\bar{M}}^{-1}\left(q\right)\tau +d,$$ (2)

where x is the end-effector position of the robot, $J\left(q\right)$ is the Jacobian matrix, and $\bar{M}\left(q\right)$ is the nominal value of the mass matrix $M\left(q\right)$ , and d is the lumped disturbance as presented as follows:

$$d=J\left(q\right)\left({\overset{˘}{M}}^{-1}\left(q\right)\tau +J^{-1}\left(q\right)\dot{J}\left(q\right)\dot{q}\right)-J\left(q\right)M^{-1}\left(q\right)\left(C\left(q,\dot{q}\right)+G\left(q\right)+\tau_{f}+\tau_{d}\right),\left(3\right)$$

where $\bar{M}\left(q\right)=\overset{˘}{M}\left(q\right)-\bar{M}\left(q\right)$ is the deviation mass matrix.

Remark 2: It is very difficult to determine accurate parameters of model (1), (2) or (3). Furthermore, the parameters sometimes vary during the working processes. To treat this drawback, the proposed controller is required to be model-free, robust and flexible.

## 3 Neural flexible PID controller

In this section, the proposed controller is designed with new features to realize the control mission stated. Theoretical effectiveness of the closed-loop system is then analyzed using Lyapunov constraints.

### 3.1 A flexible PID control framework

The controller is developed based on a conventional PID (Tan et al., 2004) structure as in Eq. 4.

$$\tau =-\bar{M}J^{+}\left(K_{p}e+K_{d}\overset{\text{.}}{e}+K_{i}\int edt\right)$$ (4)

where $e=x-x_{d}$ is the control objective, $x_{d}$ is the desired trajectory, *J* ^*+*^ is pseudo-inverse of the Jacobian *J* and $K_{p},K_{d},K_{i}$ are control gains.

We assume that the desired trajectory *x*_*d*_ is inside of the workspace of the robot and the end-effector *x* of the robot can reach to the desired position selected. Advanced path-planning and obstacle-avoidance algorithms (Mujumdar and Padhi, 2011; Shiller, 2015; Huang et al., 2019) could be employed to generate appropriate desired profiles for the robot.

In real-time control, one can tune the control gains $\left(K_{p},K_{d},K_{i}\right)$ for acceptable control performances. However, the fixed gains might not ensure good control errors for various working conditions (Thanh and Ahn, 2006), (Rocco, 1996). To cope with this problem, we propose an automatic tuning law for PID gains, as follows:

$$\left\{\dot{\begin{array}{l} K_{P}=K_{2}K_{2}+K_{1}+K_{2}{\overset{˘}{k}}_{0}\\ K_{D}=2K_{2}+{\overset{˘}{k}}_{0}\\ K_{I}=K_{2}K_{1}+{\overset{˘}{k}}_{0}K_{1}\\ {\dot{\overset{˘}{k}}}_{0}=-\alpha_{0}diag\left(\left|e\right|\right)diag{\left(1+\left|e\right|\right)}^{-1}{\overset{˘}{k}}_{0}+\beta_{0}{\left(\dot{e}+K_{2}e+K_{1}\int edt\right)}^{2} \end{array}}\right.$$ (5)

where $K_{1},\;K_{2}$ are positive core gains, $\alpha_{0}$ , $\beta_{0}$ are learning rates and ${\overset{˘}{k}}_{0}=diag\left({\overset{˘}{k}}_{0}\right)$ is the activation gain.

Remark 3: As seen in Eq. 5, the PID gains are structured from static and dynamic gains which respectively yield robustness and adaptation of the closed-loop system. The control gains are varied in non-linear manners to drive the control error to go into the desired region regardless of unknown environments. For faster control results, the disturbance term d needs to be effectively compensated by a proper control signal.

### 3.2 Additional neural network control signal

First of all, the disturbance d is modeled using the following Radial Basis Function (RBF) network:

$$d=W\xi \left(q\right)+\delta ,$$ (6)

where W is the optimal weight vector, $\xi \left(q,\;\dot{q}\right)$ is the regression vector, and δ is the modeling error.

Based on the neural network model (6), the control signal (4) is modified by adding an additional intelligent control term, as follows:

$$\tau =-\bar{M}J^{+}\left(\underset{u_{PID}}{\underset{\;}{K_{p}e+K_{d}\overset{\;}{e}+K_{i}\int edt}}+\underset{u_{NN}}{\underset{\;}{\hat{W}\xi \left(q,\dot{q}\right)}}\right)$$ (7)

where $u_{PID}$ and $u_{NN}$ stand control terms generated by PID and neural network structure, respectively, and $\hat{W}$ is estimate of the weight vector W . The estimation $\hat{W}$ is updated by the following non-linear mechanism:

$${\dot{\hat{w}}}_{i|i=1..n}=-\alpha_{w}\left|e_{i}\right|{\left(1+\left|e_{i}\right|\right)}^{-1}{\hat{w}}_{i}+\beta_{w}\left({\dot{e}}_{i}+K_{2}e_{i}+K_{1}\int e_{i}dt\right)\xi ,$$ (8)

where $\alpha_{w}$ and $\beta_{w}$ are learning rates.

Remark 4: The system (8) uses rich information including time-derivative, linear, and integral function of the control error to activate the learning process. The weight matrix of the neural network is automatically updated to ensure the minimum control error.

### 3.3 Stability analysis

In this section, we discuss the stability of the closed-loop system to ensure reliability of the proposed controller for the robotic system (3). From the above design, we have the following statements.

Theorem 1: Give a task-space model (3) of robotic manipulators, if employing a conventional neural PID control signal (7) supported by adaptive rules (5) and (8), the following properties hold:

1) The control error e , activation gain ${\overset{˘}{k}}_{0}$ and the neural weight vectors are bounded.
2) In the stationary phase, the control error e converges to zero.

Proof:

 

We first synthesize a virtual control error $\left(e_{v}\right)$ as follows:

$$e_{v}=\dot{e}+K_{2}e+K_{1}\int edt$$ (9)

The time derivative of the new error $\left(e_{v}\right)$ under dynamics (3) and the model (6) is described

$${\dot{e}}_{v}=J\left(q\right){\bar{M}}^{-1}\left(q\right)\tau +W\xi \left(q\right)+\delta -{\ddot{x}}_{d}+K_{2}\dot{e}+K{\;}_{1}e$$ (10)

By substituting the control signal Eq. 7 and the gain structure Eq. 5 into the dynamics Eq. 10, we have a simpler form:

$$\begin{array}{l} {\dot{e}}_{v}=-J\left(q\right){\bar{M}}^{-1}\left(q\right)\bar{M}J^{+}\left(\underset{u_{PID}}{\underset{\;}{K_{p}e+K_{d}\dot{e}+K_{i}\int edt}}+\underset{u_{NN}}{\underset{\;}{\hat{W}\xi \left(q,\dot{q}\right)}}\right)+W\xi \left(q,\dot{q}\right)+\delta -{\ddot{x}}_{d}+K_{2}\dot{e}+K_{1}e\\ =-K_{2}\left(\dot{e}+K_{2}e+K_{1}\int edt\right)-{\overset{˘}{k}}_{0}\left(\dot{e}+K_{2}e+K_{1}\int edt\right)-\tilde{W}\xi \left(q,\dot{q}\right)+\delta -{\ddot{x}}_{d}\\ =-K_{2}e_{v}-{\overset{˘}{k}}_{0}e_{v}-\tilde{W}\xi \left(q,\dot{q}\right)+\delta -{\ddot{x}}_{d} \end{array}$$ (11)

where $\tilde{W}=\hat{W}-W$ is estimation error of the neural weight matrix W .

We now consider a new Lyapunov function:

$$L=0.5e_{v}^{T}e_{v}+0.5{\overset{˘}{k}}_{0}^{T}{\overset{˘}{k}}_{0}+0.5\overset{n}{\underset{i=1}{\sum }}{\tilde{w}}_{i}^{T}\beta_{wi}^{-1}{\tilde{w}}_{i}.$$ (12)

Differentiating the function Eq. 12 with respect to time and noting the dynamics Eq. 11 lead to

$$\begin{array}{l} \dot{L}=e_{v}^{T}{\dot{e}}_{v}+{\overset{˘}{k}}_{0}^{T}\beta_{0}^{-1}{\dot{\overset{˘}{k}}}_{0}+\overset{n}{\underset{i=1}{\sum }}{\tilde{w}}_{i}^{T}\beta_{wi}^{-1}{\dot{\tilde{w}}}_{i}\\ =-e_{v}^{T}\left(K_{2}e_{v}+{\overset{˘}{k}}_{0}e_{v}-\delta +{\ddot{x}}_{d}\right)-\overset{n}{\underset{i=1}{\sum }}e_{vi}{\tilde{w}}_{i}^{T}\xi +{\overset{˘}{k}}_{0}^{T}\beta_{0}^{-1}\left(-\alpha_{0}diag{\left(1+\left|e\right|\right)}^{-1}{\overset{˘}{k}}_{0}+\beta_{0}e_{v}^{2}\right)\\ +\overset{n}{\underset{i=1}{\sum }}{\tilde{w}}_{i}^{T}\beta_{wi}^{-1}\left(-\alpha_{w}\left|e_{i}\right|{\left(1+\left|e_{i}\right|\right)}^{-1}{\hat{w}}_{i}+\beta_{w}\left({\dot{e}}_{i}+K_{2}e_{i}+K\;{\;}_{1}\int e_{i}dt\right)\xi \right)\\ =-e_{v}^{T}\left(K_{2}e_{v}-\delta +{\ddot{x}}_{d}\right)-{\overset{˘}{k}}_{0}^{T}\alpha_{0}diag\left(\left|e\right|\right)diag{\left(1+\left|e\right|\right)}^{-1}{\overset{˘}{k}}_{0}+\overset{n}{\underset{i=1}{\sum }}{\tilde{w}}_{i}^{T}\beta_{wi}^{-1}\left(-\alpha_{w}\left|e_{i}\right|{\left(1+\left|e_{i}\right|\right)}^{-1}\left(w_{i}+{\tilde{w}}_{i}^{T}\right)\right)\\ \leq -0.5e_{v}^{T}K_{2}e_{v}-{\overset{˘}{k}}_{0}^{T}\beta_{0}^{-1}\alpha_{0}diag\left(\left|e\right|\right)diag{\left(1+\left|e\right|\right)}^{-1}{\overset{˘}{k}}_{0}+0.5\lambda_{\max}\left(K_{2}^{-1}\right){\left\|\delta +{\ddot{x}}_{d}\right\|}^{2}-\overset{n}{\underset{i=1}{\sum }}{\tilde{w}}_{i}^{T}\beta_{wi}^{-1}\alpha_{w}\left|e_{i}\right|{\left(1+\left|e_{i}\right|\right)}^{-1}{\tilde{w}}_{i}\\ +\overset{n}{\underset{i=1}{\sum }}\beta_{wi}^{-1}\alpha_{w}\left|e_{i}\right|{\left(1+\left|e_{i}\right|\right)}^{-1}{\left\|w_{i}\right\|}^{2} \end{array}$$ (13)

Applying Cauchy-Schwarz inequality, we obtain the following result:

$$\dot{L}\leq -0.5e_{v}^{T}K_{2}e_{v}-{\overset{˘}{k}}_{0}^{T}\beta_{0}^{-1}\alpha_{0}diag\left(\left|e\right|\right)diag{\left(1+\left|e\right|\right)}^{-1}{\overset{˘}{k}}_{0}-\overset{n}{\underset{i=1}{\sum }}{\tilde{w}}_{i}^{T}\beta_{wi}^{-1}\alpha_{w}\left|e_{i}\right|{\left(1+\left|e_{i}\right|\right)}^{-1}{\tilde{w}}_{i}+∆$$ (14)

where Δ is a lumped term defined as

$$∆=0.5\lambda_{\max}\left(K_{2}^{-1}\right){\left\|\delta +{\ddot{x}}_{d}\right\|}_{\max}^{2}+\overset{n}{\underset{i=1}{\sum }}\beta_{wi}^{-1}\alpha_{w}{\left\|w_{i}\right\|}_{\max}^{2}$$ (15)

Since $w_{i}$ and δ are bounded, hence Δ is bounded as well. This discussion leads to the proof of the first statement of Theorem 1.

In the stationary phase, the time derivative of the virtual control error $e_{v}$ converges zeros. By differentiating Eq. 9 with respect to time and applying Hurwitz criterion on the results, we can achieve the second proof of Theorem 1.

**FIGURE 1:**
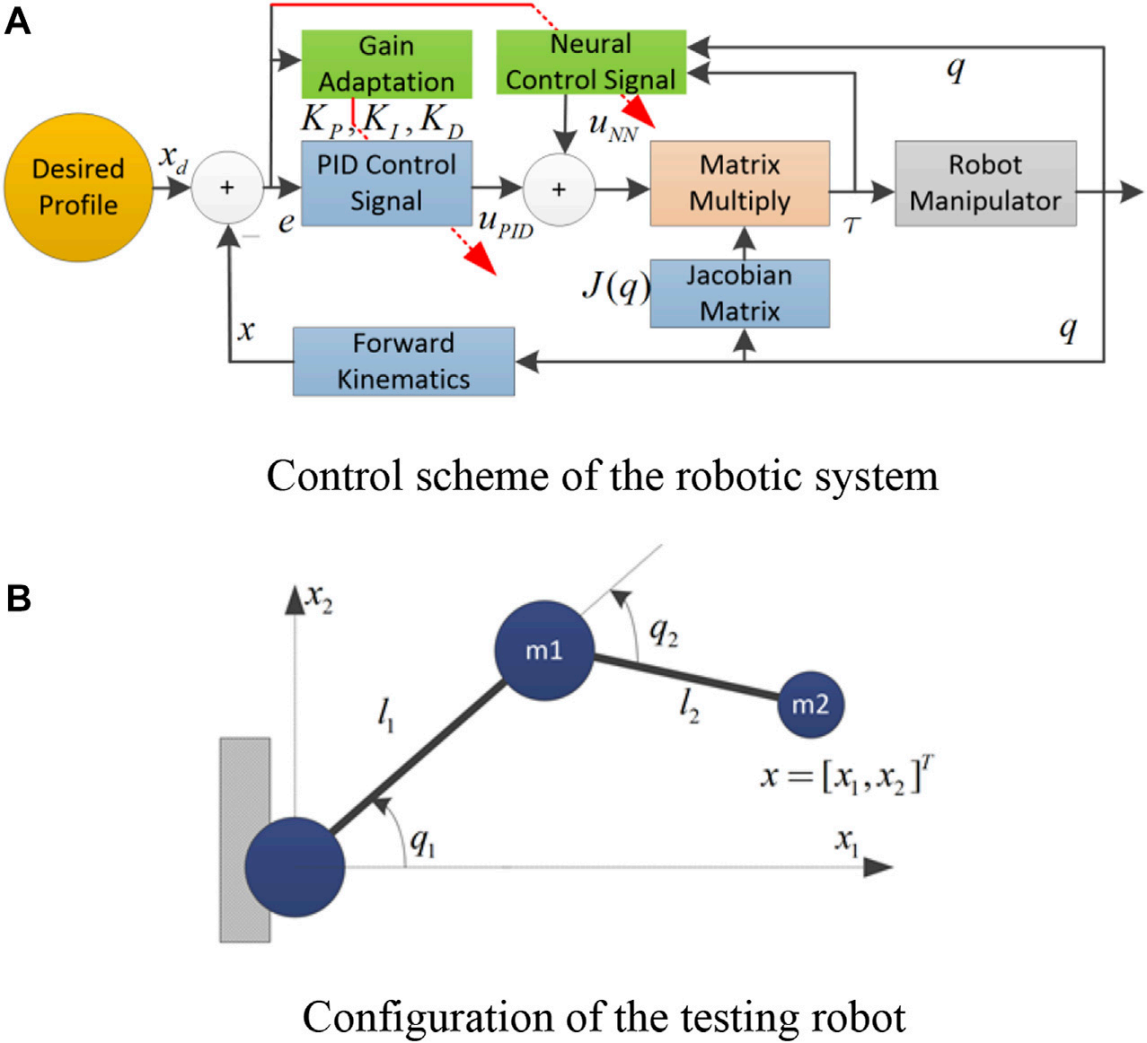
The testing robot. **(A)** Control scheme of the robotic system. **(B)** Configuration of the testing robot.

Remark 5: As carefully observing on the definition (15), one could select $K_{2}$ and $\beta_{wi}$ to large enough to reduce the disturbance bound ∆ . However, these are still fixed values. From Eq. 14, it can be seen that the control performance could be enhanced by the learning gain $k_{0}$ for various working cases. The control idea is graphically summarized in Figure 1A. The following implementation procedure could be referred for deploying the proposed control algorithm on simulation or real-time testing. 1) In the first step, all of the learning rates ( $\alpha_{0}$ , $\beta_{0}$ , $\alpha_{w}$ and $\beta_{w}$ ) are set to be zeros. The positive core gains $\left(K_{1},K_{2}\right)$ are manually tuned for acceptable control performances. The gain $K_{2}$ are recommended to be greater than the gain $K_{1}$ . 2) In the second step, the learning rates ( $\alpha_{0}$ and $\beta_{0}$ ) of the activation gain ( $K_{0}$ ) are adjusted to further enhance the control performance. In this step, the core gains $\left(K_{1},K_{2}\right)$ could be retuned in some cases for higher control precision. 3) In the third step, the regression vector $\xi \left(q,\;\dot{q}\right)$ is built and the learning rates ( $\alpha_{w}$ and $\beta_{w}$ ) of the neural network are manually selected bring the control accuracy to a higher level. The whole tuning procedure could be applied several times for seeking an excellent control outcome. Note that, from the second turn, it does not need to reset the learning rates ( $\alpha_{0}$ , $\beta_{0}$ , $\alpha_{w}$ and $\beta_{w}$ ) to be zeros anymore.

## 4 Validation results

This section presents validation results of the proposed controller in simulations. The control algorithm was applied to a 2-degree-of-freedom (DOF) robot, as sketched in Figure 1B. The manipulator was modeled as two rigid links with lengths of *l*_*1*_ and *l*_*2*_. The mass was distributed at the end of each link *(m*_*1*_ *, m*_*2*_ *)*. The robot would work in a vertical plane with downward gravitational acceleration. Viscous friction was modeled at the joints *(a*_*1*_ *, a*_*2*_ *)*. Although this robot is quite simple, it contains all the necessary components of a general multi-degree of freedom manipulator including moment of inertia, centrifugal terms, Coriolis terms, gravity terms and friction effects.

The detailed dynamic equations of the robot are as follows:

$$\{\begin{array}{l} \tau_{1}=m_{2}l_{2}^{2}\left({\ddot{q}}_{1}+{\ddot{q}}_{2}\right)+l_{1}l_{2}m_{2}\cos\left(q_{2}\right)\left(2{\ddot{q}}_{1}+{\ddot{q}}_{2}\right)+\left(m_{1}+m_{2}\right)l_{1}^{2}{\ddot{q}}_{1}\\ -m_{2}l_{1}l_{2}\sin\left(q_{2}\right){\dot{q}}_{2}\left({\dot{q}}_{2}+2{\dot{q}}_{1}\right)+m_{2}l_{2}g\cos\left(q_{1}+q_{2}\right)\\ +\left(m_{1}+m_{2}\right)l_{1}g\cos\left(q_{1}\right)+a_{1}{\dot{q}}_{1}\\ \tau_{2}=m_{2}l_{2}^{2}\left({\ddot{q}}_{1}+{\ddot{q}}_{2}\right)+l_{1}l_{2}m_{2}\cos\left(q_{2}\right){\ddot{q}}_{1}+m_{2}l_{1}l_{2}\sin\left(q_{2}\right){\dot{q}}_{1}^{2}\\ +m_{2}l_{2}g\cos\left(q_{1}+q_{2}\right)+a_{2}{\dot{q}}_{2} \end{array}.$$ (16)

To estimate the disturbances d , we used an RBF neural network with 4 input neurons, 256 hidden neurons and 2 output neurons.

The actual values of the length of links, mass and viscous friction coefficients were chosen as follows: $l_{1}=0.2;l_{2}=0.3;m_{1}=7;m_{2}=3.5;\;a_{1}=3;m_{2}=10$

To evaluate the adaptability and robustness of the controller under divergent working conditions, we compared the proposed controller (called anPID) with a conventional PID controller (referred to as cPID) and an adaptive PID controller with using only automatic tuning law for PID gains (referred to as aPID). The parameters of the controller were chosen as: $K_{1}=diag\left(\left[5;5\right]\right)K_{2}=diag\left(\left[50;50\right]\right)\bar{M}=diag\left(\left[0.1;0.1\right]\right)$ while learning coefficients were $\alpha_{0}=0.01$ , $\beta_{0}=40$ and $\alpha_{w}=0.01$ , $\beta_{w}=50$

**FIGURE 2:**
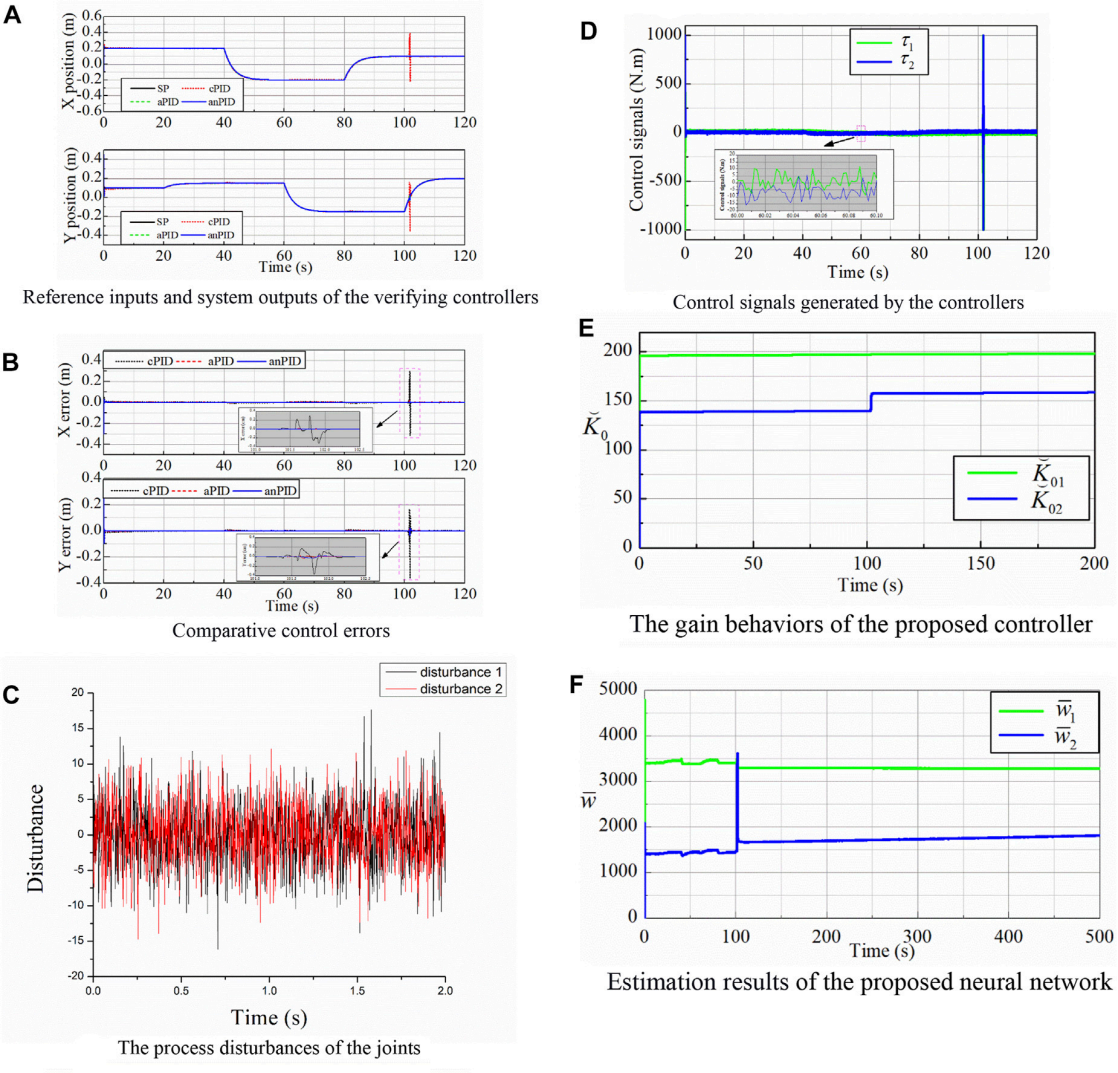
Simulation data of the controllers in the first simulation. **(A)** Reference inputs and system outputs of the verifying controllers. **(B)** Comparative control errors. **(C)** The process disturbances of the joints. **(D)** Control signals generated by the controllers. **(E)** The gain behaviors of the proposed controller. **(F)** Estimation results of the proposed neural network.

To carefully express the performance of the proposed controller, the robotic manipulators were simulated in three cases. In the first simulation, the robot was controlled to track the desired trajectories of smooth multi-step signals. Furthermore, process disturbances in the form of white noises, as shown in Figure 2C, were added to the output torques of the actuators. Simulation results of the conventional and intelligent PID controllers for the tracking control mission are also shown in Figure 2.

Figures 2A,B shows that the proposed controller maintained good control errors even though the end-effector of the manipulator worked throughout a singularity point of (0.1; 0) (m). Figure 2D exhibits the control signals of the smart PID controller which had large values at the initial and singularity points in order to decrease the control errors as fast and much as possible. This superior property was the achievement of the learning laws (5) and (8) that are demonstrated by the gain and weight variations as depicted in Figures 2E, F, respectively. These terms were first started from the zero value, then their values had a large overshoot to bring the system to the steady state rapidly. It can be seen that the system adapted to the reasonable approximation of the disturbances to bring the control error to the smallest possible value. Therefore, the learning ability of the system has been confirmed with uncertain non-linearities and perturbations through this simulation validation.

**FIGURE 3:**
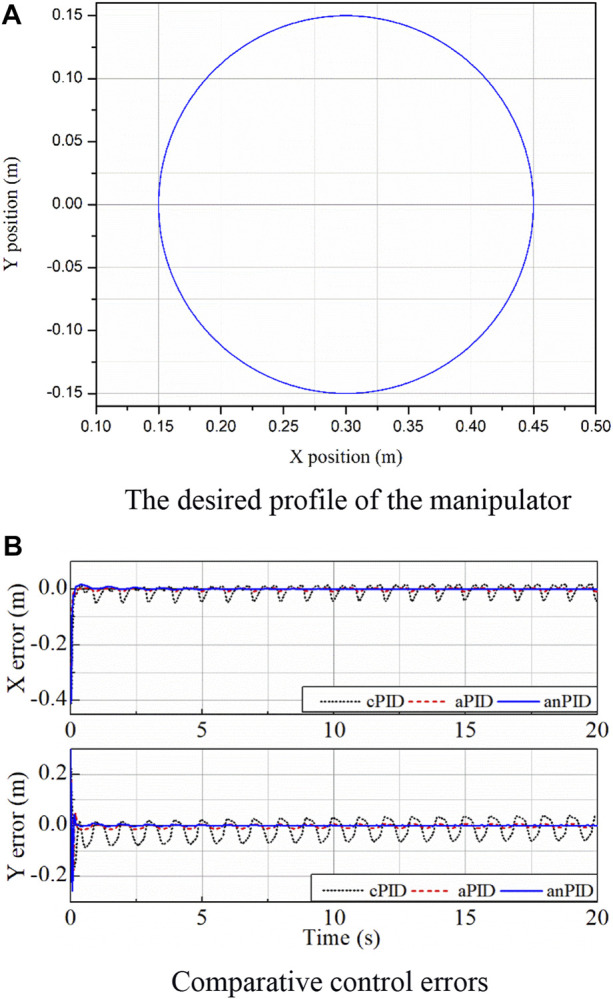
Simulation data of the controllers in the second simulation. **(A)** The desired profile of the manipulator. **(B)** Comparative control errors.

The manipulator was employed to draw a circle whose radius was 0.15 m and origin was at a point of (0.3; 0) (m) with a frequency of 1 Hz in the second simulation. The reference input used is shown in Figure 3A.

With the application of the neural flexible PID controller for unknown environments but using the adaptive rule (7), the control results obtained are presented in Figure 3B. From the data in this figure, although disturbances were not known in advance, the control qualities of the joints were good at both the transient and steady-state phases. The results were achieved thanks to the learning characteristics of the PID gains and the designed RBF neural network. There was a little overshoot in the *y*-direction error due to the large learning rate selected, but this overshoot might cause the system to quickly reach steady state. From the comparison of the control data in Figure 3B, it can be seen that the quality of proposed controller (anPID) was better than that of the aPID controller which was employed only one learning law (5). This is possible because the more adaptive terms the controller had, the more approximation with disturbances it gained.

**FIGURE 4:**
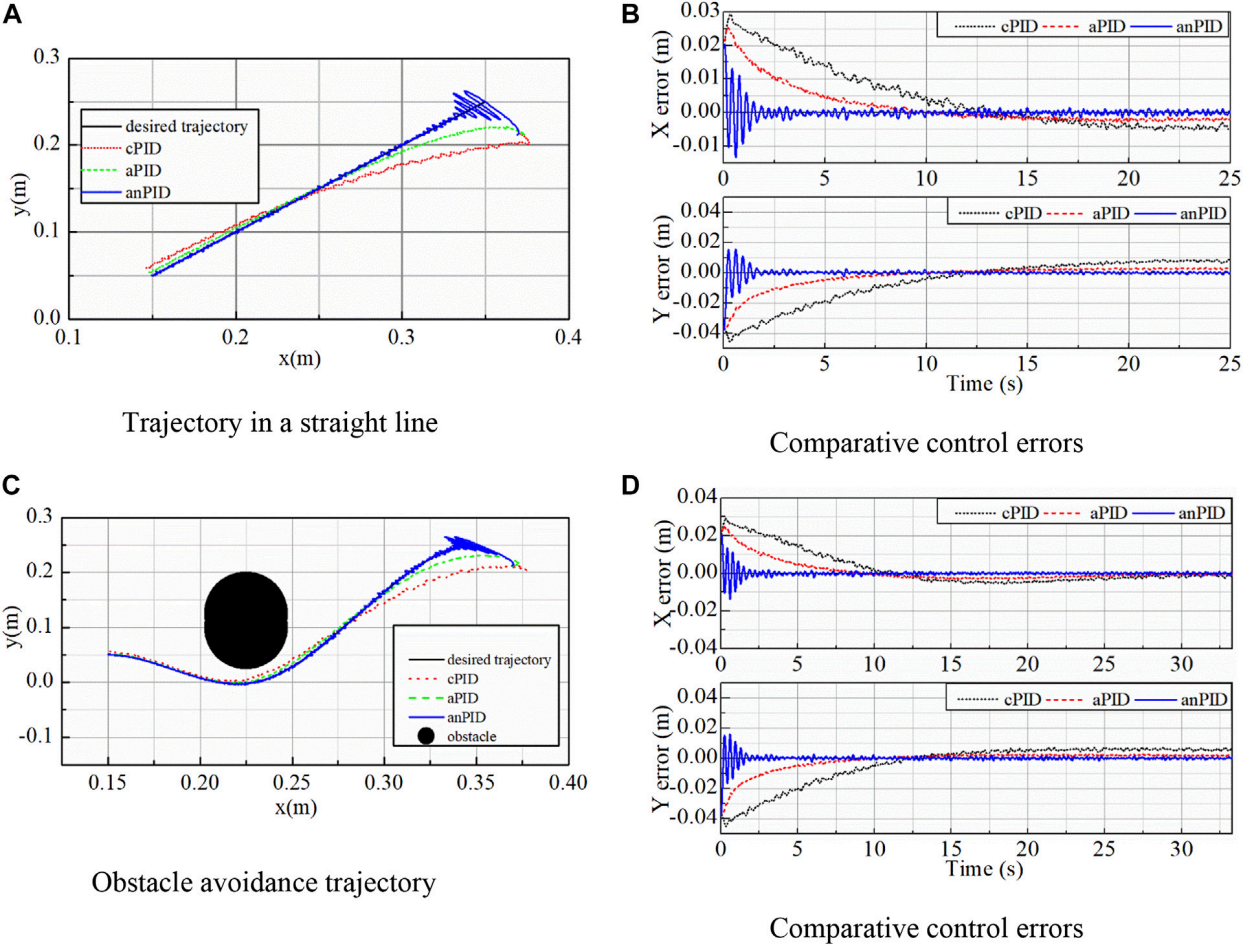
Simulation data of the controllers in the third simulation. **(A)** Trajectory in a straight line. **(B)** Comparative control errors. **(C)** Obstacle avoidance trajectory. **(D)** Comparative control errors.

In the third simulation, the end effector of robot manipulator was controlled to move from a point of (0.35; 0.25) (m) to another point of (0.15; 0.05) (m). After applying the three controllers for this mission in a free condition in which the desired trajectory was planned as a straight line, their control outcomes including the actual outputs and the control errors were illustrated in Figures 4A, B, respectively. In these figures, although the proposed controller (anPID) had more oscillation in the transient state to find adaptive term quickly, it had smallest overshoot and steady state error when compared with cPID and aPID controllers.

To further challenge the controllers with a more difficult working condition, an obstacle was set on the moving trajectory of the robot in the task space. By applying the trajectory planning method and the referred avoidance collision method (Borenstein and Koren, 1991), (Craig, 2005), the desired trajectory was generated as a curve by using two third-order-segment polynomials for the position, velocity and acceleration of the end-effector. The control data in this case are shown in Figures 4C, D. From the comparison of the data in these figures, it can be seen that the control quality of proposed controller (anPID) was better than that of the others (aPID and cPID) even though with the non-linear trajectory generated.

Table 1 described the maximum absolute (MA) and root-mean-square (RMS) values of the control performances for a specified manipulated time (20 s–25 s). The proposed controller always provided the best MA and RMS error in all cases. These results show that the proposed control technology compensated efficiently for the non-linear uncertainties and unknown disturbances. Here, the advantages of the proposed controller have been confirmed. Therefore, the simulation results have proved that the studied control method outperform over the previous ones.

**TABLE 1:** Statistical computation of the controllers from the validation results.

Control error		X position		Y Position	
		MA	RMS	MA	RMS
The 1st case	cPID	0.0045	0.0023	0.0036	0.0021
	aPID	0.0019	9.36 × 10^−04^	0.0016	7.66 × 10^−4^
	anPID	7.28 × 10^−4^	2.45 × 10^−4^	7.16 × 10^−4^	2.27 × 10^−4^
The 2nd case	cPID	0.0423	0.0189	0.059	0.0362
	aPID	0.0089	0.0046	0.0088	0.0059
	anPID	7.33 × 10^−4^	3.14 × 10^−4^	0.0016	4.91 × 10^−4^
The 3rd case (no obstacle)	cPID	0.0055	0.0044	0.0089	0.0077
	aPID	0.0029	0.0021	0.0033	0.0027
	anPID	0.0013	5.24 × 10^−4^	0.0012	5 × 10^−4^
The 3rd case (obstacle)	cPID	0.0048	0.0037	0.0072	0.0057
	aPID	0.0026	0.0018	0.0029	0.0022
	anPID	8.37 × 10^−4^	3.14 × 10^−4^	0.0016	6.9 × 10^−04^

## 5 Conclusion

In this paper, an intelligent controller is proposed to optimize the position control performance of a 2DOF robotic manipulator. The controller is developed based on a conventional PID structure. New advanced features designed for disturbance learning and gain adaptation are then integrated into the ordinary control signal to improve its robustness and result in high control accuracies. The control efficiency of the proposed approach was then successfully verified by theoretic proof and comparative simulations. It can confirm that the controller is model-free, simple, robust and flexible. In the near future, the proposed control algorithm will be integrated with an additional control term that could result in asymptotic control performances for dynamical trajectories. Furthermore, advanced path-planning and obstacle-avoidance algorithms will be considered to combine with the controller to increase the flexibility when the system works in complex environments.

## Data Availability

The raw data supporting the conclusion of this article will be made available by the authors, without undue reservation.

## References

[B1] Astrom K. Hagglund K. (1995). PID controllers: Theory, design and tuning. Washington, DC, USA: ISA Press.

[B2] Ba D. X. Bae J. B. (2020). A nonlinear sliding mode controller of serial robot manipulators with two-level gain-learning ability. IEEE Access 8, 189224–189235. 10.1109/access.2020.3032449

[B3] Ba D. X. Bae J. B. (2021). A precise neural-disturbance learning controller of constrained robotic manipulators. IEEE Access 9, 50381–50390. 10.1109/access.2021.3069229

[B4] Ba D. X. Tran M. S. vu V. P. Tran V. D. Tran M. D. Tai N. T. (2021). “A neural-network-based nonlinear controller for robot manipulators with gain-learning ability and output constraints,” in 2021 International Symp. Electrical and Electronics Engineering (ISEE), Ho chi minh, Vietnam, 149–153.

[B5] Ba D. X. Yeom H. Bae J. B. (2019). A direct robust nonsingular terminal sliding mode controller based on an adaptive time-delay estimator for servomotor rigid robots. Mechatronics 59. May. 10.1016/j.mechatronics.2019.03.007

[B6] Berscheid L. Kroeger T. (2021). “Jerk-limited real-time trajectory generation with arbitrary target states,” in Proceedings of Robotics: Science and Systems, July 2021.

[B7] Bledt G. Powell M. J. Katz B. Carlo F. D. Wensing P. W. Kim S. (2018). MIT cheetah 3: Design and control of a robust, dynamic quadruped robot in 2018 IEEE/RSJ International Conference on Intelligent Robots and Systems (IROS), Madrid, Spain.

[B8] Borenstein J. Koren Y. (1991). The vector field histogram-fast obstacle avoidance for mobile robots. IEEE Trans. Robot. Autom. 7 (3), 278–288. 10.1109/70.88137

[B9] Boscario P. Gasparetto A. Vidoni R. (2012). “Planning continuous-jerk trajectories for industrial manipulators,” in Proceedings of the ASME 2012 11th Biennial Conference on Engineering Systems Design and Analysis, Nantes, France, July 2012.

[B10] Craig J. J. (2018). Introduction to robotics: Mechanics and control. 4. Hoboken, NJ, USA: Pearson Prentice Hall. th ed.

[B11] Craig J. J. (2005). Manipulator dynamic Introduction to robotics: Mechanics and control,. 3. Hoboken, NJ, USA: Pearson Prentice Hall, 165–200. inrd ed.s

[B12] Cucientes M. Moreno D. L. Bugarin A. Barro S. (2007). Design of a fuzzy controller in mobile robotics using genetic algorithms. Appl. Soft Comput. 7 (2), 540–546. 10.1016/j.asoc.2005.05.007

[B13] Gao X. Li X. Sun Y. Hao L. Yang H. Xiang C. (2022). Model-free tracking control of continuum manipulators with global stability and assigned accuracy. IEEE Trans. Syst. Man. Cybern. Syst. 52 (2), 1345–1355. 10.1109/tsmc.2020.3018756

[B14] He W. Sun Y. Yan Z. Yang C. Li Z. Kaynak O. (2020). Disturbance observer-based neural network control of cooperative multiple manipulators with input saturation. IEEE Trans. Neural Netw. Learn. Syst. 31 (5), 1735–1746. 10.1109/tnnls.2019.2923241 31425054

[B15] Huang S. Teo R. S. H. Tan K. K. (2019). Collision avoidance of multi unmanned aerial vehicles: A review. Annu. Rev. Control 48, 147–164. 10.1016/j.arcontrol.2019.10.001

[B16] Juang C. F. Chang Y. C. (2011). Evolutionary-group-based particle-swarm-optimized fuzzy controller with application to mobile-robot navigation in unknown environments. IEEE Trans. Fuzzy Syst. 19 (2), 379–392. 10.1109/tfuzz.2011.2104364

[B17] Karayiannidis Y. Papageorgiou D. Doulgeri Z. (2016). A model-free controller for guaranteed prescribed performance tracking of both robot joint positions and velocities. IEEE Robot. Autom. Lett. 1 (1), 267–273. 10.1109/lra.2016.2516245

[B18] Kim D. H. Cho J. H. (2006). A biological inspired intelligent PID controller tuning for AVR systems. Int. J. Control, Automation, Syst. 4 (5), 624–636.

[B19] Minguez J. Montano L. (2004). Nearness diagram (ND) navigation: Collision avoidance in troublesome scenarios. IEEE Trans. Robot. Autom. 20 (1), 45–59. 10.1109/tra.2003.820849

[B20] Mujumdar A. Padhi R. (2011). Reactive collision avoidance of using nonlinear geometric and differential geometric guidance. J. Guid. Control Dyn. 34 (1), 303–311. 10.2514/1.50923

[B21] Neath M. J. Swain A. K. Madawala U. K. Thrimawithana D. J. (2014). An optimal PID controller for a bidirectional inductive power transfer system using multiobjective genetic algorithm. IEEE Trans. Power Electron. 19 (3), 1523–1531. 10.1109/tpel.2013.2262953

[B22] Park H. W. Park S. Kim S. (2015). “Variable-speed quadrupedal bounding using impulse planning: Untethered high-speed 3D Running of MIT Cheetah 2,” in 2015 IEEE International Conference on Robotics and Automation (ICRA), Seattle, USA. in.,

[B23] Rocco P. (1996). Stability of PID control for industrial robot arms. IEEE Trans. Robot. Autom. 12 (4), 606–614. 10.1109/70.508444

[B24] Shiller Z. (2015). Off-line and on-line trajectory planning Motion and operation planning of robotic systems, mechanisms and machine science. Switzeland: Springer International Publishing, 29–62.

[B25] Tan G. Z. Zeng Q. D. Li W. B. (2004). Intelligent PID controller based on ant system algorithm and fuzzy inference and its application to bionic artificial leg. J. Cent. South Univ. Technol. 11, 316–322. 10.1007/s11771-004-0065-7

[B26] Thanh T. D. C. Ahn K. K. (2006)., 16. Mechatronics. 10.1016/j.mechatronics.2006.03.011 Nonlinear PID control to improve the control performance of 2 axes pneumatic artificial muscle manipulator using neural network

[B27] Wang M. Wang Z. Chen Y. Sheng W. (2020). Adaptive neural event-triggered control for discrete-time strict-feedback nonlinear systems. IEEE Trans. Cybern. 50 (7), 2946–2958. 10.1109/tcyb.2019.2921733 31329140

[B28] Wensing P. M. Wang A. Seok S. Otten A. Lang J. Kim S. (2017).Proprioceptive actuator design in the MIT cheetah: Impact mitigation and high-bandwidth physical interaction for dynamic legged robots, IEEE Trans. Robot, 33. IEEE Transactions on Robotics, 509–522. 10.1109/tro.2016.2640183

[B29] Ye J. (2008). Adaptive control of nonlinear PID-based analog neural networks for a nonholonomic mobile robot. Neurocomputing 71. 10.1016/j.neucom.2007.04.014

[B30] Zhu W. H. (2010). Virtual Decomposition Control: Toward hyper degrees of freedom robots. Berlin Heidelberg: Springer-Verlag.

